# Preliminary Dose Constraints for Testing Intensity Modulated Proton Therapy in Anal Cancer Trials

**DOI:** 10.1016/j.ijpt.2026.101971

**Published:** 2026-10-18

**Authors:** Rahul Krishnatry, Aditi Jain, Rohidas Punde, Shakthivel Mani, Shivakumar Gudi, Reena Engineer

**Affiliations:** 1 Tata Memorial Centre, HBNI University, Mumbai, India; 2 Advanced Centre for Treatment, Research and Education in Cancer, Tata Memorial Centre, HBNI University Mumbai, Mumbai, India

**Keywords:** Proton therapy, Anal canal cancers, IMPT, Dose constraints

## Abstract

**Purpose:**

The target volume for radiotherapy of anal cancer is complex, with the added challenge of it being surrounded by several critical organs at risk (OARs). In this study, we review the dosimetric advantage that can be achieved with intensity modulated proton therapy (IMPT) over intensity modulated radiotherapy (IMRT) and derive the dose constraints for OARs.

**Materials and Methods:**

Ten consecutive patients of anal cancer treated using rotational IMRT (Rapid Arc; Varian Medical Systems, Palo Alto, USA) in 2023 were reviewed. The contours of the target volumes and OARs (bowel, bladder, marrow, bilateral femoral heads, and external genitalia) were reviewed on Eclipse (v16.1) treatment planning system for adherence to contouring guidelines. The clinical plans were re-evaluated for OAR doses and target volume coverage.

The scans with structure sets were exported to RayStation treatment planning system (v12A) for intensity modulated proton therapy (IMPT) planning. The dose for IMPT planning was prescribed to the CTV with 5 mm robustness, keeping dose prescription similar to the clinical IMRT plan (45-50 Gy to pelvis and 55-60 Gy simultaneous boost to any gross disease and gross nodes). The 4 beam class solution was identified as best for IMPT planning. The IMPT plan objective was set 10% to 20% below the lower threshold of RTOG 05–29 and UK guidelines, followed by principles of ALARA for OARs without compromising target coverage. The dose-volume parameters, conformity index (CI) and homogeneity index (HI) were compared for IMRT versus IMPT using student’s *t*-test.

**Results:**

Target volume coverage was well achieved with both IMRT and IMPT. However, V100% of GTV_p_ and GTV_n_ and V95%, V98%, V99%, V100% of CTV_Pelvis_ and CTV_TumourBoost_ were significantly better with IMPT. HI and CI of target volumes were also significantly improved with IMPT. There was a significant decrease in volume of OARs receiving doses <40 Gy. Volumes receiving higher doses (40-50 Gy) were very small and not significantly affected with use of IMPT.

**Conclusion:**

IMPT offers improved target coverage with reduction in low-intermediate dose spill to OARs. Based on these results, dose constraints for IMPT planning have been generated for future clinical trial validation.

## Introduction

Carcinoma of the anal canal represents a rare subset of gastrointestinal cancers, comprising roughly 1% to 2% of all digestive tract neoplasms.^1^, ^2^ Over recent decades, the incidence of anal cancer has risen globally, particularly among populations with HPV exposure and immunodeficiency, highlighting the need for continual refinement of therapeutic strategies.^1^, ^2^, ^3^

Definitive chemoradiotherapy remains the cornerstone of management, offering high rates of local control and sphincter preservation.^4^, ^5^, ^6^, ^7^, ^8^ Photon-based intensity-modulated radiotherapy (IMRT) has improved target conformity and normal tissue sparing compared to older techniques such as 3-dimensional conformal radiation therapy (3D-CRT), leading to reduced acute toxicity.^9^, ^10^ Nevertheless, treatment-related morbidity including acute hematologic suppression, gastrointestinal and genitourinary toxicity remain a significant challenge. These toxicities affect over 80% of patients cumulatively and may compromise treatment compliance, with frequent and prolonged breaks ultimately may impact overall outcomes.^9^, ^10^, ^11^, ^12^

Proton beam therapy, due to its unique physical properties characterized by the Bragg peak and rapid dose fall-off beyond the target, allows for superior dose conformity with substantially lower exit doses to adjacent organs at risk (OAR).^13^, ^14^ These dosimetric advantages have generated growing interest in evaluating proton therapy for pelvic malignancies, particularly in anatomically complex sites such as the anal canal. Simultaneous dose sparing of bone marrow, small bowel, bladder, femora and genitalia is challenging with photons.

Preliminary planning studies and early clinical reports suggest that proton beam therapy reduces low-dose spill to OARs while maintaining a similar high-dose distribution as IMRT.^15^, ^16^, ^17^, ^18^ However, many of these studies applied dose constraints originally designed for IMRT. It seems that most centers relied on RTOG photon IMRT parameters without fully exploring the true potential of protons by defining constraints specific to proton therapy. In addition, the use of large planning target volumes (PTVs) and sequential robustness margins may have further expanded prescription volumes, increasing overlap between high-dose regions and OARs. Collectively, these factors might have diminished the realization of the true potential clinical benefit for proton therapy.

In the current study, we re-planned previously treated photon IMRT patients using proton IMPT to identify the best potential dose parameters that could be achieved and derive the constraints for future prospective randomized trial (CTRI/2024/05/066687, NCT06630793) and clinical validation.

## Materials and methods

After obtaining approval from the institutional review board (IEC No. 4336), ten consecutive patients diagnosed with squamous cell carcinoma of the anal canal with clinical stage T2–4b N0–2a M0, who received radical radiotherapy between 1st January, and 31st December 2023 were identified and included in the study.

### Photon intensity-modulated radiotherapy planning

The patients were already planned and treated cases of Photon-based IMRT. The IMRT planning process is described in short here for completeness.

All patients were immobilized in the supine position using a knee rest with arms raised above the head. A contrast-enhanced Computed Tomogram scan was taken from diaphragm to mid-thigh with an axial slice thickness of 2.5 mm. Bladder protocol (500 ml water over 30 minutes after emptying bladder) was followed in all patients at simulation and each treatment.

Gross tumor volumes (GTVp, GTVn) were delineated in Eclipse v16.1 (Varian Medical Systems Inc, Palo Alto, USA) treatment planning system using clinical findings and diagnostic or planning T2-weighted MRI axial sequence as available. A uniform 5 mm expansion to the GTVp and GTVn was given to generate the clinical target volume (CTVp, CTVn) and the CTV pelvis was contoured as per UK Consensus guidelines.^19^ The PTV were obtained by a uniform 5 mm expansion from the respective CTVs. The OARs were delineated on the planning CT namely bladder, bowel bag, bilateral femora, external genitalia and pelvic bone marrow (Supplementary Figure 1) based on RTOG Guidelines.^9^ IMRT plans were generated using 2 or more rotational arcs to achieve the optimal class solution (Figure 1a). The RTOG and UK guidelines along with principle of ALARA were generally followed while plan evaluation and acceptance.^9^, ^19^ Most patients were treated to a dose of 45 to 50 Gy to pelvis and 55 to 60 Gy to gross disease as simultaneous integrated boost.

**Figure 1:**
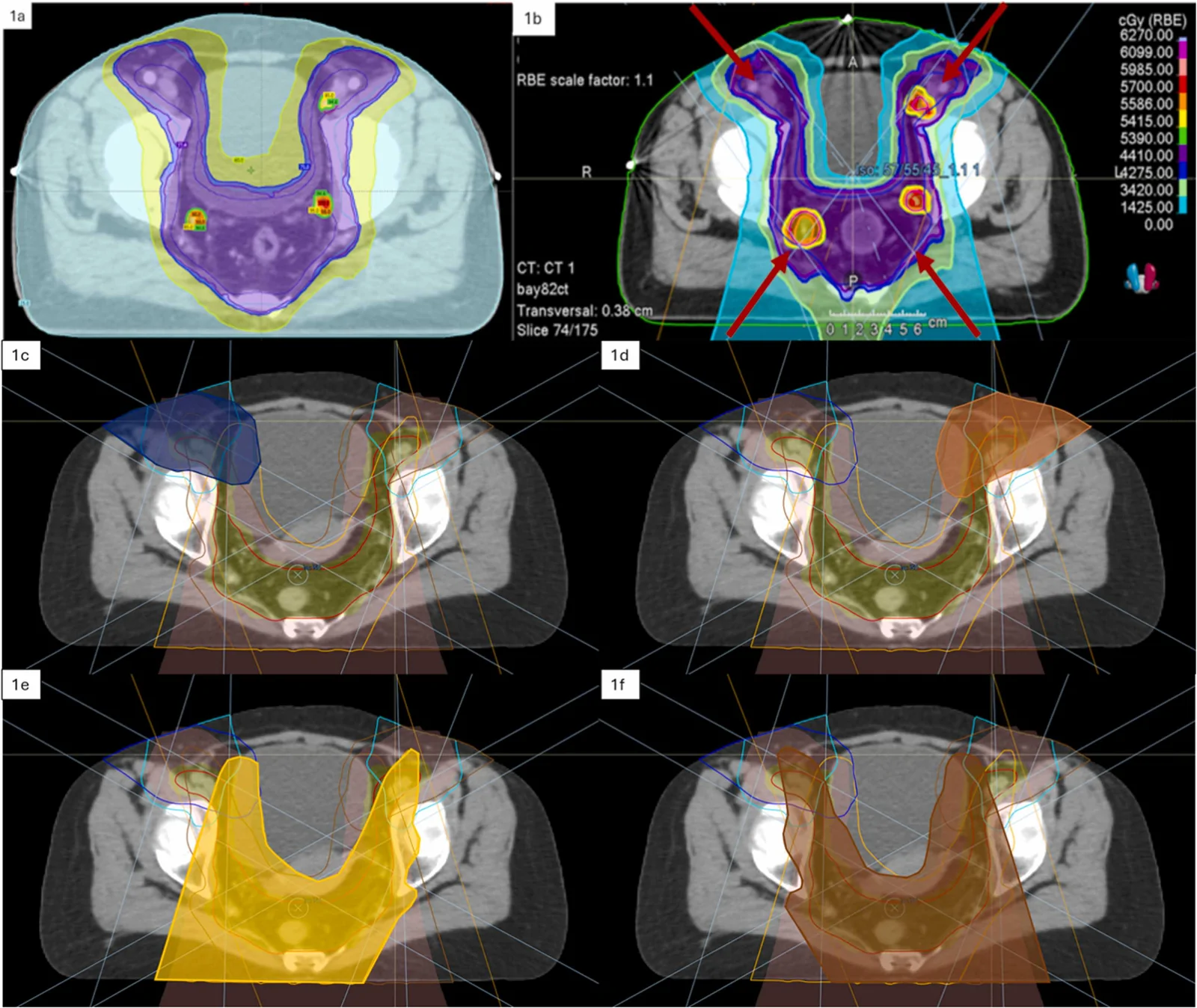
Dose distribution of (a) IMRT and (b) IMPT plans of a patient demonstrating a decrease in low dose spill. The direction of the beams in the IMPT plan are depicted by the red arrows in (b). The legend for the isodoses of both plans is shown in (b), where the dose contribution by (c) right anterior oblique in navy blue, (d) left anterior oblique in yellow, (e) right posterior oblique in orange, and (f) left posterior oblique in brown.

For this study, the contours and treated plans were reviewed on the Eclipse treatment planning system for adherence to contouring guidelines,^20^ dose coverage and OAR sparing and were found to be suitable as per RTOG and UK guidelines.^9^, ^19^

### Proton intensity modulated proton therapy planning

The planning CT images and associated structure sets, including the GTV, CTV, and OARs, were imported into RayStation (RaySearch Laboratories, Stockholm, Sweden). For IMPT planning, robustness margins to CTVs were employed instead of photon-based PTV_photons. All transferred contours were reviewed for geometric integrity, including the presence of sharp edges or contour distortions, and corrected where necessary. IMPT plans were generated based on a standardized institutional protocol using the same dose prescription as the clinically delivered IMRT plans, with a constant relative biological effectiveness value of 1.1. Intravenous contrast regions were delineated and assigned the appropriate tissue density overrides. The dose was prescribed to the CTV using robustness settings of 5 mm setup error and 3.5% density uncertainty for IMPT optimization.^18^, ^21^, ^22^, ^23^

We reviewed all previously published class solutions for anal cancer IMPT^18^, ^23^, ^24^, ^25^ and identified the 4-beam class solution as the best (Figure 1b). Robust plans were generated by a trained medical physicist using 4-field beam geometry. The right anterior oblique primarily covered the right inguinal region and the right external iliac nodes (Figure 1c) while the right posterior oblique covered the gross primary disease, the right internal iliac nodes and the right obturator nodes (Figure 1e). Similarly, the left anterior oblique primarily covered the left inguinal region and the left external iliac nodes (Figure 1d) while the left posterior oblique covered the gross primary disease, the left internal iliac nodes and the left obturator nodes (Figure 1f). Sometimes an additional sub beam to anterior obliques or a direct posterior beam was added on a case-to-case basis to achieve most optimal dose distribution. Beam specific optimization target volumes were created to avoid the beams passing through critical structures (especially gastrointestinal tract). Despite these measures, when a bowel loop was present within the beam path or encompassed within the target volume, it was individually contoured and anatomical robust optimization was done (air: 0.001 gm/cc and water: 1 gm/cc).^26^ However, in clinical practice, it might be helpful to verify this by acquiring a CT scan during the course of treatment.

The initial IMPT plan objective was set at 10% to 20% lower threshold for RTOG 05–29 and UK guidelines, followed by principles of ALARA for OARs namely bladder, bowel bag, bilateral femora and external genitalia, without compromising target coverage. For target coverage, the plan objectives were to cover 98% of the target volume by 98% prescription dose in nominal plan and 98% of the target volume by 95% prescription dose in robust plan.

In our planning protocol, priority was given to adequate target coverage while maintaining an acceptable level of robustness against range and setup uncertainties. OAR constraints were considered concurrently; however, target coverage and robustness were prioritized when trade-offs were necessary. If OAR constraints could not be met without compromising target coverage, further optimization/replanning was performed. Where OAR constraints remained unachievable, limited target undercoverage was accepted, provided that ≥98% of the target received ≥95% of the prescribed dose in the nominal plan and ≥95% received ≥95% in the robust plan.

## Statistical methods

The clinical and dosimetric data of the 10 patients included in the study were entered into an excel sheet. The dose-volume parameters, conformity index (CI = V_RI_/TV where RI or reference isodose volume is defined by ICRU as V_95%_ and TV is the target volume) and homogeneity index (HI = D_2%_/D_98%_) were noted for IMRT versus IMPT.

The paired differences between IMPT and IMRT methods for each dose-volume parameter were assessed with the student’s *t* test. A *P* value < .05 was considered statistically significant.

### Development of dosimetric constraints

The dosimetric constraints were derived as follows – mean+ SD was taken as the optimal constraint (preliminary) and mean + 2SD was taken as the mandatory thresholds to be achieved in prospective patients in our institutional clinical practice and further clinical trial (NCT06630793). The clinical correlation of achieving these constraints versus deviations would be considered with that data being generated in future.

## Results

The median age of the patients was 56 years (Range: 39-69 years) with a male: female ratio of 7:3. All patients had a Karnofsky Performance status score of 80 to 100 prior to the start of radiotherapy. The distribution of T stage was T2:T3:T4 = 31%:46%:23% with 77% patients being node positive (Table 1).

**Table 1:** General characteristics and treatment details.

Characteristic		No. of patients
Age - Median (IQR)		56 (39-61.5)
Gender	Male	7
	Female	3
Histology	Squamous cell carcinoma	10
T-Stage	cT2	3
	cT3	5
	cT4	2
N-Stage	cN0	1
	cN1a/b	8
	cN2	1
M-Stage	cM0	10
	cM1	0
KPS	100	3
	80-90	7
	≤70	0
EBRT Dose	45Gy/25# + SIB to gross nodes and primary 55 Gy	2
	45Gy/25# + SIB to gross nodes 55 Gy; to primary 57 Gy	8
Chemo Regimen	Capecitabine + Mitomycin-C	9
	Single agent 5-Fluorouracil	1
Skin Toxicity	Grade ≥ 3	3
	Grade 1-2	6
Gastrointestinal Toxicity	Grade ≥ 3	1
	Grade 1-2	4
Genitourinary Toxicity	Grade ≥ 3	0
	Grade 1-2	4
Hematological Toxicity	Grade ≥ 3	0
	Grade 1-2	3

### Target coverage

The median prescription dose for PTV_Pelvis_ = 45 Gy, PTV_NodalBoost_ = 55 Gy and PTV_TumourBoost_ = 57 Gy. The target volume coverage was well achieved with both modalities. However, the V100% isodose coverage of the GTV_p_ and GTV_n_ with proton therapy planning was significantly better with protons (*P* = .023 and 0.046 respectively). Similarly, V95%, V98%, V99%, V100% of the CTV_Pelvis_ and CTV_TumourBoost_ were significantly better with IMPT (Table 2). However, V105% was similar with both IMPT and IMRT (*P* = .916).

**Table 2:** Doses achieved – IMRT vs IMPT – target volumes.

Structure	Parameter	Photon mean	Proton mean	Absolute difference	% Difference	*P* value
GTVp	D95%	99.48%	99.64%	0.15	0.153	.758
	D98%	98.85%	99.24%	0.39	0.397	.507
	D99%	87.77%	98.99%	11.22	12.784	.12
	D100%	74.70%	96.47%	21.77	29.143	.023
	CI	0.99	1.00	0.15	0.15	.76
GTVn	D95%	53.85%	53.85%	0.00	0.055	.916
	D98%	53.55%	53.62%	0.00	0.131	.836
	D99%	51.56%	53.38%	−0.02	3.533	.146
	D100%	40.82%	53.30%	−0.12	30.558	.046
	CI	0.54	0.54	0.00	0.05	.92
CTV_Pelvis	V95%	97.75%	99.98%	−2.23	2.282	.000
	V98%	90.16%	99.59%	−9.43	10.459	.000
	V99%	82.75%	98.16%	−15.41	18.621	.000
	V100%	72.37%	91.74%	−19.37	26.770	.000
	CI	0.98	1.00	−0.223	2.282	.000
	HI	1.47	1.14	−32.93	22.410	.048
CTV_NodalBoost	V95%	97.50%	100.00%	2.50	2.564	.102
	V98%	84.29%	99.71%	15.42	18.297	.080
	V99%	74.69%	97.92%	23.24	31.111	.063
	V100%	61.14%	89.44%	28.29	46.276	.079
	CI	0.97	1.0	0.03	3.093	.049
	HI	110.63%	104.85%	−5.79	5.229	.047
CTV_TumourBoost	V95%	98.88%	99.98%	−1.10	1.111	.012
	V98%	91.96%	99.65%	−7.69	8.362	.004
	V99%	82.57%	98.16%	−15.59	18.880	.027
	V100%	67.99%	86.80%	−18.81	27.665	.024
	V105%	0.08%	0.08%	0.01	6.364	.916
	CI	0.99	1.00	−1.10	1.111	.012
	HI	1.07	1.04	2.70	2.522	.001

Overall, IMPT achieved significantly better HI (Pelvis = 1.47 vs 1.14; Nodal boost = 1.11 vs 1.05; Tumor boost = 1.07 vs 1.04, with *P* = .048, .047 and .001, respectively) and CI (Pelvis = 0.98 vs 1.0, *P* < .001; Nodal boost = 0.97 vs 1.0, *P* = .049; Tumor boost = 0.99 vs 1.0, *P* = .012) than the corresponding photon plans (Table 2).

### Organs at risk

There was a 44.71% reduction in the volume of the bowel receiving 30 Gy (*P* = .03) and 37.03% reduction in V35Gy (*P* = .05). However, the reduction in higher doses received by the bowel was not significant (Figure 2a; Table A1). A similar pattern was also noted for the bladder, with an absolute reduction of 17.33% in V35Gy (*P* = .009). There was an absolute reduction by 9.77% in V40Gy and 1.62% in V50Gy to the bladder, which were not significant (Figure 2b; Table A1).

**Figure 2:**
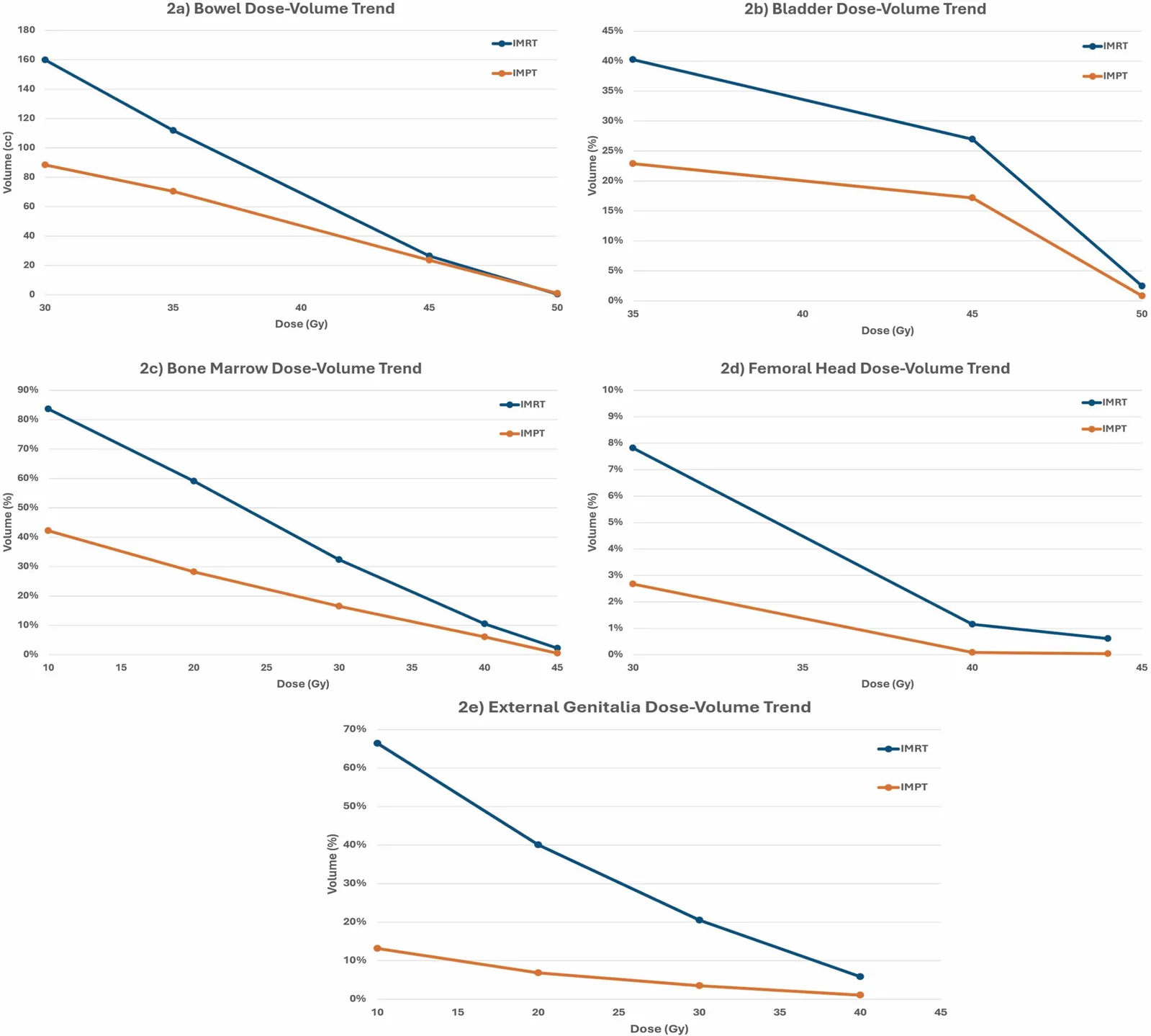
Graphical comparison of dose-volume trends of OARs with IMRT and IMPT: (a) bowel, (b) bladder, (c) bone marrow, (d) femoral head, and (e) external genitalia. The orange line depicts the dose-volume trend for IMPT planning, and the blue line depicts the dose-volume trend for IMRT planning in the graphs.

The absolute reduction in V10Gy, V20Gy, and V30Gy to the bone marrow by 41.51%, 30.77% and 15.78% respectively, was significant (with *P* < .001 for each). The reduction in V40Gy and V45Gy was only 4.52% and 1.73% (*P* = .1 and .06, respectively). The D_mean_ to bone marrow for IMRT plans was 24.27 Gy which was reduced to 12.3 Gy, that is, a 11.97 Gy reduction (*P* < .001) (Figure 2c; Table A1).

There was a significant decrease in the volume of femoral heads receiving 30 Gy by 5.14% (*P* = .01). The reduction in V40Gy and V44Gy by 1.07% and 0.57% was not statistically significant (*P* = .3 each) (Figure 2d; Table A1). There was a 53.17% absolute reduction in the volume of the external genitalia receiving 10 Gy, with a 33.28% absolute reduction in V20Gy and 17% for V30Gy (*P* < .01 for all). As was observed for the other OARs, the decrease by 4.7% in V40Gy was not significant (*P* = .08) (Figure 2e; Table A1).

The volumes receiving higher doses (40-50 Gy) were not significantly different for any of the OARs. This was probably because the volumes receiving these doses by either modality, were very small (IMRT: IMPT - Bowel - V45Gy = 26.23:23.4cc & V50Gy = 0.36:0.8cc; Bladder V50Gy = 2.43:0.81%; Marrow - V40Gy = 10.48:5.96% & V45Gy 2.13:0.40%; Femoral heads - V40Gy = 1.15:0.08% & V44Gy = 0.6:0.03%; External genitalia - V40Gy = 5.79:1.05%). This was due to very minimal overlap between target and OARs volumes.

### Dose constraints

Dose constraints for OARs and target coverage are detailed in Table 3, Table 4 with a side-by-side comparison with the corresponding IMRT constraint as per RTOG or UK Consensus Guidelines.^9^, ^19^

**Table 3:** Planning objectives for target coverage – IMRT vs IMPT.

Structure	Parameter	Mean	Standard deviation (SD)	Optimal/preliminary (Mean + SD)	Mandatory (clinical trial) (mean + 2 SD)	Photon constraint
GTVp	D95%	99.6%	0.51%	99%	98.5%	-
	D98%	99.2%	0.51%	98.5%	98%	-
	D99%	98.9%	0.57%	98.5%	98%	-
	D100%	96.5%	1.03%	95%	90%	-
GTVn	D95%	96.4%	1.1%	95.5%	94%	-
	D98%	96.0%	1.0%	95%	93.5%	-
	D99%	95.6%	1.0%	94.5%	93%	-
	D100%	95.1%	1.3%	93.5%	92.5%	-
CTV_Pelvis	V95%	99.6%	0.02%	99.95%	99.92%	>95%^(19)^
	V98%	98.2%	0.30%	99.00%	98.50%	-
	V99%	91.7%	1.22%	96.19%	94.05%	-
	V100%	99.9%	4.34%	84.84%	77.22	>85%^(9)^
	V105%	99.59%	8.68%	16.63%	1.4%	-
CTV_Nodal Boost	V95%	100%	0.00%	100%	100%	>95%^(19)^
	V98%	99.71%	0.15%	100%	100%	-
	V99%	97.92%	0.98%	99.87%	100%	>90%^(19)^
	V100%	89.44%	5.77%	98.63%	100%	>90%^(9)^
CTV_Tumour Boost	V95%	99.98%	0.02%	99.96%	99.93%	>95%^(19)^
	V98%	99.65%	0.13%	99.5%	99.27%	-
	V99%	98.16%	0.83%	96.99%	95.54%	>90%^(19)^
	V100%	86.80%	5.50%	79.28%	69.63%	>90%^(9)^
	V105%	0.08%	0.60%	1.45%	2.50%	<5%^(19)^

**Table 4:** Dose Constraints for OARs – IMRT vs IMPT.

Structure	Parameter	Mean	Standard deviation (SD)	Optimal/preliminary (mean + SD)	Mandatory (clinical trial) (mean + 2 SD)	Photon constraint
Bowel	V30Gy	88.3cc	42.5cc	130cc	170cc	<200cc^(9)^
	V35Gy	70.4cc	36.0cc	110cc	140cc	<150cc^(9)^
	V45Gy	23.4cc	13.8cc	37cc	50cc	<20cc^(9)^
	V50Gy	0.8cc	2.3cc	3cc	5cc	5cc^(19)^
Bladder	V35Gy	22.90%	2.97%	26%	30%	<50%^(9)^
	V40Gy	17.14%	2.72%	20%	25%	<35%^(9)^
	V50Gy	0.81%	1.34%	3%	5%	<5%^(9)^
Bone Marrow	V10Gy	42.16%	6.25%	50%	55%	-
	V20Gy	28.21%	4.52%	35%	40%	-
	V30Gy	16.50%	3.13%	20%	25%	<50%^(9)^
	V40Gy	5.96%	2.66%	10%	15%	<35%^(9)^
	V50Gy	0.40%	0.36%	1%	5%	<5%^(9)^
	Dmean	12.3 Gy	1.4 Gy	13.5 Gy	15.0 Gy	-
Femoral Head	V30Gy	2.67%	7.47%	10%	20%	<50%^(9)^
	V40Gy	0.08%	0.17%	0.5%	5%	<35%^(9)^
	V44Gy	0.03%	0.10%	0.1%	1%	<5%^(9)^
External Genitalia	V10Gy	13.15%	7.58%	20%	30%	-
	V20Gy	6.79%	4.48%	10%	20%	<50%^(9)^
	V30Gy	3.43%	2.45%	5%	10%	<35%^(9)^
	V40Gy	1.05%	0.93%	2%	5%	<5%^(9)^

## Discussion

The dosimetric comparison demonstrates that IMPT can substantially improve the dose distribution to potentially improve therapeutic ratio in anal canal. Not only did IMPT consistently outperform IMRT in target coverage metrics, but this was also supplemented by a reduction in low–intermediate range doses to OARs. The volume of overlap of OARs with target volumes for both IMRT and IMPT was small, suggesting that the gastrointestinal, genitourinary, dermatologic, and hematologic toxicities were more likely to be attributable to the low and intermediate dose spill to these organs.^27^ This strengthens the rationale for IMPT in anal malignancies.

Clinically, this dosimetric advantage provides a powerful mechanism to reduce treatment-limiting acute toxicities. Specifically, the significant reduction in pelvic marrow irradiation aims to lower the high rates of grade 3+ myelosuppression traditionally associated with pelvic chemoradiation.^28^ Furthermore, the near-elimination of unnecessary dose to the external genitalia and mid-dose bowel exposure offers a strong rationale for decreasing severe acute dermatitis, proctitis, and long-term genitourinary morbidity.

As reported by Wo et al, long-term outcomes of patients treated with definitive chemoradiation using proton therapy for anal cancer are comparable to IMRT with a significant reduction in grade 2+ toxicities but no dosimetric correlation with late toxicities.^29^ In this study, dose was prescribed to CTV with a robustness margin resulting in smaller overlap volumes. This could potentially result in a significant correlation between OAR-dosimetry and treatment outcomes.

The study helped develop and standardize the planning and dose constraints for IMPT in anal cancer for institutional clinical practice and future clinical trial. The optimal constraints will act as starting point for planning while mandatory constraints will be more relevant for deviations. Further prospective clinical correlation would derive more meaningful information in the future. If clinical use of proton therapy is able to decrease toxicity burden, dose escalation may also be tested in future to improve outcomes.

The limitations of the current study include small retrospective nature of the study and the pending dosimetric uncertainty due to the impact of GI gas on tissue-density and absence of clinical correlation. Furthermore, the IMPT dose distribution can be offset by its unique vulnerability to uncertainties in proton range, patient setup, anatomical variations, and motion requiring robust planning, adaptive strategies, and vigilant quality assurance.^30^

## Conclusion

IMPT offers superior low-intermediate dose sparing to OARs versus IMRT, alongside enhanced target coverage, homogeneity, and conformity for target volumes in anal canal squamous cell carcinoma. These dosimetric gains warrant IMPT evaluation in high-risk patients prone to hematologic, gastrointestinal, or genitourinary toxicity during chemoradiation, pending validation through prospective clinical trials assessing toxicity and outcomes. The dose constraints derived from this study will be used for treatment planning of patients enrolled in a future prospective randomized controlled trial (NCT06630793).

## Ethics declaration

The authors have NOT obtained informed consent from participants or their legal representatives. The requirement for individual patient informed consent was waived by the IEC because this is a retrospective dosimetric analysis using previously collected, fully anonymized patient CT datasets for treatment replanning and comparison. This involved no active patient intervention, and all personal identifiers were removed prior to data analysis to maintain patient confidentiality.

This study was performed in compliance with relevant laws, regulatory frameworks and guidelines where the research took place. This study was approved by the Tata Memorial Center (Approval No. OIEC/4336/2024/00002).

## Ethical Approval

This study was reviewed and approved by the Institutional Review Board of Tata Memorial Centre, Mumbai (IEC No. 4336, 26 March 2024).

## CRediT authorship contribution statement

**Rahul Krishnatry:** Conceptualization, Formal analysis, Methodology, Project Administration, Supervision, Validation, Visualization, Writing – review and editing; **Aditi Jain:** Conceptualization, Data curation, Formal analysis, Methodology, Validation, Visualization, Writing – original draft, Writing – review and editing; **Rohidas Punde:** Conceptualization, Data curation, Methodology, Validation, Writing – review and editing; **Shakthivel Mani:** Conceptualization, Methodology, Writing – review and editing; **Shivakumar Gudi:** Conceptualization, Writing – review and editing; **Reena Engineer:** Conceptualization, Writing – review and editing.

## Declaration of Conflicts of Interest

The authors declare that they have no known competing financial interests or personal relationships that could have appeared to influence the work reported in this paper.

## Data Availability

Data supporting this study are included within the article. The derived data supporting the findings of this study are available upon reasonable request from the corresponding author, Rahul Krishnatry, MD.

## Footnotes

Appendix A Supplementary data associated with this article can be found in the online version at doi:10.1016/j.ijpt.2026.101971.
