# Supplementary material for: Preliminary Dose Constraints for Testing Intensity Modulated Proton Therapy in Anal Cancer Trials
Source: Int J Part Ther. 2026 Oct 18;22:101971. doi: 10.1016/j.ijpt.2026.101971 (PMC13742319; doi:10.1016/j.ijpt.2026.101971)
Supplement: Supplementary file 1 [file mmc1.docx]

**Table A.1: Doses achieved – IMRT vs IMPT – OARS**

| **Structure** | **Parameter** | **Photon Mean** | **Proton Mean** | **Absolute Difference** | **% Difference** | **p Value** |
| --- | --- | --- | --- | --- | --- | --- |
| **Bowel** | V30Gy | 159.707 | 88.3cc | -71.407 | 44.71 | 0.031 |
|  | V35Gy | 111.805 | 70.4cc | -41.405 | 37.03 | 0.058 |
|  | V45Gy | 26.232 | 23.4cc | -2.832 | 10.80 | 0.925 |
|  | V50Gy | 0.362 | 0.8cc | 0.438 | 120.99 | 0.087 |
| **Bladder** | V35Gy | 40.23% | 22.90% | -17.33 | 43.08 | 0.009 |
|  | V40Gy | 26.91% | 17.14% | -9.77 | 36.31 | 0.066 |
|  | V50Gy | 2.43% | 0.81% | -1.62 | 66.67 | 0.072 |
| **Bone Marrow** | V10Gy | 83.67% | 42.16% | -41.51 | 49.61 | 0.000 |
|  | V20Gy | 58.98% | 28.21% | -30.77 | 52.17 | 0.000 |
|  | V30Gy | 32.28% | 16.50% | -15.78 | 48.88 | 0.000 |
|  | V40Gy | 10.48% | 5.96% | -4.52 | 43.13 | 0.115 |
|  | V45Gy | 2.13% | 0.40% | -1.73 | 81.22 | 0.062 |
|  | Dmean | 24.27 | 12.3Gy | -11.97 | 49.32 | 0.000 |
| **Femoral Head** | V30Gy | 7.81% | 2.67% | -5.14 | 65.81 | 0.013 |
|  | V40Gy | 1.15% | 0.08% | -1.07 | 93.04 | 0.297 |
|  | V44Gy | 0.60% | 0.03% | -0.57 | 95.00 | 0.355 |
| **External Genitalia** | V10Gy | 66.32% | 13.150% | -53.17 | 80.17 | 0.000 |
|  | V20Gy | 40.07% | 6.79% | -33.28 | 83.05 | 0.000 |
|  | V30Gy | 20.43% | 3.43% | -17.0 | 83.22 | 0.003 |
|  | V40Gy | 5.79% | 1.05% | -4.74 | 81.87 | 0.083 |
